# Timing of gestational diabetes diagnosis, gestational weight gains and offspring growth trajectory: a prospective birth cohort study

**DOI:** 10.1186/s12884-023-05954-2

**Published:** 2023-09-07

**Authors:** Xiao-guang Yin, Peng Wang, Mei-ting Zhou, De-qin Li, Rui-xue Tao, Fang-biao Tao, Yang Wang, Peng Zhu

**Affiliations:** 1 Department of Pediatrics, First Affiliated Hospital of Anhui Medical University, Hefei, China; 2 Present Address: Department of Neonatology, Hefei Women and Child Health Care Hospital, Hefei, China; 3 Department of Maternal, Child & Adolescent Health, School of Public Health, Anhui Medical University, 81 Meishan Road, Hefei, China; 4 MOE Key Laboratory of Population Health Across Life Cycle, Hefei, China; 5 NHC Key Laboratory of Study on Abnormal Gametes and Reproductive Tract, Hefei, China; 6 Anhui Provincial Key Laboratory of Population Health and Aristogenics, Hefei, China; 7 Department of Disinfection and Sterilization, Hefei Centers for Disease Control and Prevention, Hefei, China; 8 Department of Nephrology, High-tech Zone, First Affiliated Hospital of Anhui Medical University, Hefei, China; 9 Department of Obstetrics and Gynecology, the First People’s Hospital of Hefei City, Hefei, China

**Keywords:** Gestational diabetes mellitus, Gestational weight gain, Pregnancy, Growth trajectory, Obesity

## Abstract

**Background:**

The evidence on the associations of the timing of maternal gestational diabetes mellitus (GDM) with the comprehensive growth trajectory from perinatal to early childhood in offspring is limited. The potential mechanism remains elusive. Our aim is to estimate the associations of the timing of GDM diagnosis and gestational weight gains (GWG) with the growth trajectory of children from perinatal to early childhood.

**Methods:**

A total of 7609 participants are included from the Maternal & Infants Health in Hefei cohort study. Primary predictors were the timing of maternal GDM diagnosis and GWG during pregnancy. The main outcomes included fetal ultrasonic measurements, birth size as well as BMI peak indicators during infancy within 48 months.

**Results:**

GDM diagnosed before 26 weeks was associated with increased risks of overgrowth for fetal abdominal circumference (OR 1.19, 95% CI 1.04–1.36) and birth weight (OR 1.51, 95% CI 1.19–1.91) when compared with unexposed. GDM diagnosis < 26 weeks was related to the higher BMI peak (β 0.16, 95%CI 0.03–0.28) within 48 months. The significantly additive impacts of maternal early GDM diagnosis and excessive gestational weight gains (EGWG) on offspring overgrowth were observed. Women in GDM < 26 weeks with early EGWG group had higher levels of hsCRP compared with GDM > 26 weeks (*P* < 0.001).

**Conclusions:**

Exposure to maternal GDM diagnosed before 26 weeks with early EGWG could lead to shifts and/or disruptions from the typical growth trajectory from perinatal to early childhood in offspring.

**Supplementary Information:**

The online version contains supplementary material available at 10.1186/s12884-023-05954-2.

## Introduction

Gestational diabetes mellitus (GDM) is one of the most frequent metabolic disorders during pregnancy, which mainly leads to fetal overgrowth [1]. Exposure of fetuses to maternal GDM had increased risks of large for gestational age (LGA) and childhood obesity [2, 3]. The Pregnancy Outcome Prediction (POP) study found that accelerated fetal growth could be identified prior to the diagnosis of GDM [4]. In a small-scale longitudinal study, children exposed to maternal GDM by 26 weeks’ gestation have higher waist-to-height ratios during childhood, a marker of abdominal adiposity, but there were no significant changes in BMI or body fat compared with unexposed children [5]. Although the screening for GDM was recommended during 24–28 weeks’ gestation, these evidence implied the relationship between the timing of GDM diagnosis as a surrogate of the timing of exposure to hyperglycemia and adverse outcomes in offspring. In addition, investigations on the association of the timing of GDM diagnosis and the growth trajectory from perinatal to childhood were limited and those studies did not include the assessment of infancy BMI peak, the milestone of early life body BMI trajectories [6]. It is now well established from a variety of studies that the infancy BMI peak was a strong risk factor for childhood obesity and metabolic dysfunction [7, 8].

The timing of excessive gestational weight gain (EGWG) was a known risk factor for GDM and was a potential predictor of adverse outcomes for infants. A cohort study demonstrated that infants whose mothers with EGWG in the first half of pregnancy were characterized as excessive body fat at birth [9]. There was increasing evidence that early higher GWG during pregnancy was associated with later obesity and cardio-metabolic risk in offspring [10, 11]. It is currently unknown whether the timing of GDM diagnosis and EGWG have additive impacts on the growth trajectory in offspring.

Extensive research has indicated low-grade chronic inflammation as the biological mechanism linking maternal diabetes with adverse developmental outcomes in offspring [12, 13]. Nonetheless, data addressing the association of inflammatory response with neonatal adiposity outcomes produced controversial results [13, 14]. There has been no detailed investigation of estimating the role of low-grade chronic inflammation in the relationship among the timing of GDM diagnosis, EGWG and excessive fetal growth.

Intrauterine exposure to maternal GDM affect the development and function of the placenta (such as impaired trophoblast invasion and spiral artery remodeling) through increased inflammation and oxidative stress, dyslipidemia, maternal-fetal macronutrient transport dysfunction and altered hormone levels [15–17]. These abnormal placental changes could lead to poor fetal growth outcomes (i.e., macrosomia). A small-scale case-control study found that placental proangiogenic VEGF and pro-inflammatory CD31 expression in women with GDM were higher than those without GDM, and VEGF as well as CD31 expression was associated with GWG and increased pre-pregnancy BMI [18].

Therefore, the present prospective birth cohort aimed to investigate the association of the timing of GDM diagnosis and EGWG with the trajectory of fetal, infant, and early childhood growth. Given the fact that high-sensitivity C-reactive proteins (hsCRP) was identified as a biomarker of systemic and low-grade chronic inflammation and was applied in clinical practice, we tested the role of maternal hsCRP level in this association.

## Methods

### Participants

From March 2015 to June 2020, the participants are derived from the Maternal & Infants Health in Hefei (MIH-Hefei) cohort study in 3 centers including Anhui Women and Child Health Care Hospital, The First People’s Hospital of Hefei City, and The First Affiliated Hospital of Anhui Medical University. Women with age ≥ 18y, no communication problems, residents of Hefei, and singleton pregnancy were recruited. We have access to data for characteristics of pregnant women and infants based on medical records as well as a standardized questionnaire in the second and third trimesters and at delivery. In addition, anthropometric data for the infants at 0, 3, 6, 9, 12, 18 and 24 months (mo) were obtained in the follow-up survey. Written informed consent was obtained from all pregnant women and the study was approved by the Ethics Committee of Anhui Medical University (number: 2,015,002).

### Assessment of GWG and maternal GDM during pregnancy

In our study, total GWG was calculated as last measured weight before delivery minus pre-pregnancy weight; early GWG was determined to be the difference between weight between 16 and 20 weeks and pre-pregnancy weight; late GWG was calculated as the difference between last weight measured before delivery and body weight measured at 16–20 weeks of gestation [9]. GWG rate was calculated based on the difference in weight, divided by the number of gestational weeks in that interval. The early EGWG was defined as measured weight at study entry–pre-pregnancy weight–(2.0 kg+[X *{week of gestation at study entry–12}]), where 2.0 kg is the maximum recommended weight gain in the first trimester (up to 12 weeks of gestation) and X is the maximum recommended weekly weight gain based on the maternal pre-pregnancy body mass index (BMI) status and the 2009 IOM GWG recommendations: 0.45 to 0.59 kg/week (pre-pregnancy BMI: <18.5 kg/m^2^), 0.36 to 0.45 kg/week (pre-pregnancy BMI: 18.5–23.9 kg/m^2^), 0.23 to 0.32 kg/week (pre-pregnancy BMI 24.0-27.9 kg/m^2^), and 0.18 to 0.27 kg/wk (pre-pregnancy BMI > 28 kg/m^2^) [19]. The late EGWG was defined as last measured weight before delivery–measured weight between 16 and 20 weeks – (week of gestation of last weight before delivery–week of gestation at study entry)*X, where X is the recommended weekly weight gain.

Pregnant women completed a 75-g oral-glucose-tolerance test (OGTT) and the diagnosis of GDM was based on the criteria proposed by the International Association of Diabetes in Pregnancy Study Groups (IADPSG): fasting plasma glucose (FPG) ≥ 5.1 mmol/L, and/or 1 h plasma glucose (1-h PG) ≥ 10.0 mmol/L, and/or 2 h plasma glucose (2-h PG) ≥ 8.5 mmol/L [20]. Women were divided into three groups: unexposed (non-GDM), GDM diagnosed < 26 weeks, and GDM diagnosed ≥ 26 weeks. These cutoffs of gestational age of GDM diagnosis for the GDM group were rounded to the median weeks of the OGTT tests for all participants and the nearest week for clinical relevance [5, 21]. The cutoffs also provided reasonable sample sizes in each stratum to have appropriate statistical power to assess overgrowth risk in offspring. Women have at least one risk factors for hyperglycemia including previous gestational diabetes, pre-pregnancy higher than 30 kg/m^2^, age ≥ 40 years, first-degree relative with diabetes, previous macrosomia, or polycystic ovary syndrome, which prompted doctors to advice for an early OGTT test.

### The measurement of maternal hsCRP level

Blood samples collection at 32–36 weeks was performed by a trained technician (nurse) after 8–12 h of overnight fasting, samples were centrifuged at 3000 rpm for 5 min to retrieve plasma and instantly frozen at − 80 °C. The levels of maternal hsCRP were measured by using the enzyme-linked immunosorbent assay (ELISA) kits (Cusabio Biotech, Wuhan, China) according to the manufacturer’s instructions. Above 90th percentile hsCRP level was defined as the higher inflammatory status in this study. The minimum detectable concentration was 0.02 µg/ml, and the inter-class and intra-class coefficients of variation were less than 10%.

### Offspring growth assessment

The repeated ultrasound measurements of bi-parietal diameter (BPD), abdominal circumference (AC), and femur length (FL) were obtained with two scans, second trimester scans take place before the diagnosis of GDM (14–28 weeks) to screen for fetal anomalies, and third trimester scans (≥ 28 weeks) are conducted for obstetric indications such as a breech presentation or in uterine growth retardation. Since the two complete ultrasound records of each woman cannot be obtained, we restricted our analyses to pregnant women who had at least one ultrasound measurement. The z-scores and percentile concerning gestational age and sex of anthropometric data of fetuses and infants were calculated using the international standard developed by the Intergrowth-21st Project [22, 23]. Overgrowth was defined as a sex- and gestational age- specific Z-score percentile > 90th in this study.

To estimate infant BMI peak, children who had completed a minimum of six measurements of weight and length within 48 mo of age were included in the analysis and anthropometric data for the infants were obtained from clinic measurements. The modeling approach that was used to derive infant BMI peak characteristics in this study has been described in detail [24–26]. We fitted individual BMI curves using mixed-effects models with natural cubic spline functions, to capture the non-linear trend in BMI. Random effects were estimated to account for repeated measures in the same child and to assess the nonlinear trend in BMI. Constraints were used for an increase of stability of the curve by fixing the spline to be linear prior to 0.1 months and after 48 months. The optimum number and location of knots were calculated by the Bayesian information criterion. We estimated BMI peak characteristics (age, magnitude at peak and prepeak BMI velocity) by differentiating each child-specific BMI curve.

### Confounding variables

All potential covariates were selected a priori. Potential confounders in this study included maternal age (years), education level (junior high school and below, high school or junior college, bachelor’s degree and above), household monthly income (< 4000, 4000–8000, ≥ 8000 yuan/month), pre-pregnancy BMI (kg/m^2^), parity (nulliparous or multiparous), total GWG rate (kg/week), family history of diabetes (yes or no), infant sex (male or female), and mode of delivery (vaginal delivery/caesarean section). Moreover, women’s lifestyle factors (the frequency of physical exercise, the supplement of folic acid, calcium as well as iron) during pregnancy were taken into account in the analysis.

### Statistical analysis

Demographic characteristics were compared among groups using variance analysis and Kruskal - Wallis tests for continuous data as well as chi-square test for categorical data. Considering the subject-specific effect of repeated measures across time during pregnancy, we used mixed effect models. In the analyses of fetal ultrasound parameters, we fitted the linear mixed models to handle repeated measurements to calculate the mean change and the 95% confidence interval (CI) of the Z-scores of fetal ultrasound measurements which were associated with the time of GDM diagnosis, early GWG rate and hsCRP. Then the generalized linear mixed models (mixed effects logistic regression) were fitted to calculate the odds ratio (OR) and 95% *CI* of the associations of gestational age at GDM diagnosis, early EGWG and high hsCRP with the risk of fetal overgrowth.

Multiple linear regression models were used to analyze the associations of gestational age at GDM diagnosis, early GWG rate and hsCRP with Z scores of birth size parameters and infant BMI peak. Multiple logistic regression models were used to estimate the interrelationships between gestational age at GDM diagnosis, early EGWG, the levels of hsCRP and the risks of overgrowth of newborns. In addition, these two models were applied in determining the associations between the combined status of the time of GDM diagnosis and early GWG with the development of offspring. All regression models were adjusted for the variables listed in the covariates section. All data analyses were performed using R version 4.0.5 and SPSS statistical software (Statistical Package for the Social Sciences version 23.0, IBM Corp: Armonk, NY, USA).

## Results

### Participants

Participants with the following characteristics were excluded in the study: diagnosed with liver or renal dysfunction (n = 87), preexisting diabetes (n = 20), assisted conception (n = 112), unavailable OGTT data (n = 134) and gestational hypertension (n = 115). Finally, 7609 women were included in our study (Figure S1). Of the 7609 pregnant women, the median weeks of all participants with GDM diagnosis were 26 weeks (180 days), 951 (12.5%) were exposed to GDM diagnosed before 26 weeks (median: 22.0 weeks), and 547 (7.2%) were exposed to GDM diagnosed after 26 weeks (median: 29 weeks) (Figure S2). There were no significant differences in maternal age, pre-pregnancy BMI, household income, parity, weekly GWG (early, late and total), family history of diabetes, delivery gestational age and mode of delivery among these groups (Table 1). Specifically, compared with children whose mothers with late diagnosis of GDM, children whose mothers with early diagnosis of GDM had a higher EGWG during pregnancy (0.33 ± 0.18 VS 0.30 ± 0.14, kg/week, *P* = 0.010). There were no significant differences in the characteristics between the participants and non-participants in the study (Table S1). Meanwhile, we excluded participants who had no records of data of outcomes, and whose records were considered as unreasonable [values below or above 3 standard deviations(SD)], which yielded a final sample size of 6540 fetuses, 6008 newborns and 5183 infants (Figure S1).

**Table 1:** Characteristics of the study population

Characteristics	Non-GDM (N = 6111)	Timing of GDM diagnosis		^a^*P* value	^b^*P* value
		< 26 weeks (N = 951)	≥ 26 weeks (N = 547)		
**Maternal**					
Age, y	28.8(4.17)	30.14(4.58)	30.69(4.64)	< 0.001	0.018
Pre-pregnancy BMI, kg/m^2^	21.36(2.84)	22.31(3.18)	22.58(3.23)	< 0.001	0.129
Education ≤ 12 y	4586(75.0)	719(75.6)	417(76.2)	0.278	0.784
Household income < 4000 yuan	1143(18.7)	194(20.4)	115(21.0)	0.017	0.774
Multiparous	3230(52.9)	435(45.7)	226(41.3)	< 0.001	0.097
Early GWG rate, kg/wk	0.28(0.12)	0.35(0.14)	0.31(0.12)	< 0.001	0.009
Late GWG rate, kg/wk	0.50(0.28)	0.45(0.32)	0.38(0.31)	< 0.001	0.325
Family history of diabetes	493(8.1)	144(15.1)	89(16.3)	< 0.001	0.562
Physical exercise ≥ 3days/wk	2741(44.9)	396(41.6)	234(42.8)	0.135	0.667
Folic acid supplement < 1 times/d	4206(68.8)	673(70.8)	369(67.5)	0.354	0.180
Calcium supplement < 1 times/d	3364(55.0)	530(55.7)	312(57.0)	0.639	0.623
Iron supplement < 1 times/d	560(9.2)	83(8.7)	49(9.0)	0.904	0.880
Fiber (n/week)	18.3(3.7)	18.0(3.9)	18.0(3.8)	0.016	0.999
Protein (n/week)	12.1(4.0)	13.0(3.7)	13.0(3.9)	< 0.001	0.987
Fat (n/week)	16.0(4.3)	15.1(4.3)	14.9(4.3)	< 0.001	0.998
Carbohydrates (n/week)	7.3(3.2)	6.9(3.0)	6.8(2.9)	< 0.001	0.979
FPG, mmol/L	4.45 ± 0.33	5.17 ± 0.52	5.10 ± 0.54	< 0.001	0.003
1 h-PG, mmol/L	7.04 ± 1.34	9.30 ± 1.79	9.26 ± 1.89	< 0.001	0.654
2 h-PG, mmol/L	6.18 ± 0.98	7.87 ± 1.45	7.82 ± 1.43	< 0.001	0.388
**Infant**					
Birth weight, g *	3410(445)	3450(499)	3447(483)	0.044	0.741
Delivery gestational age, week *	39.42(1.31)	39.17(1.43)	39.13(1.35)	< 0.001	0.555
Male sex *	2527(52.4)	390(54.0)	251(54.4)	0.530	0.066
Vaginal delivery *	3234(67.0)	455(63.0)	287(62.3)	0.019	0.085
BMI at birth, kg/m^2^	13.5(1.2)	13.7(1.3)	13.8(1.3)	< 0.001	0.646
BMI at 3 months, kg/m^2^	17.3(1.5)	17.3(1.5)	17.3(1.5)	0.989	0.998
BMI at 6 months, kg/m^2^	17.8(1.5)	17.8(1.5)	17.8(1.5)	0.808	0.998
BMI at 9 months, kg/m^2^	17.6(1.5)	17.5(1.5)	17.6(1.4)	0.693	0.999
BMI at 12 months, kg/m^2^	17.1(1.3)	17.1(1.3)	17.2(1.3)	0.987	0.987
BMI at 18 months, kg/m^2^	16.4(1.2)	16.3(1.2)	16.0(1.2)	0.688	0.969
BMI at 24 months, kg/m^2^	16.0(1.2)	16.0(1.2)	15.9(1.2)	0.452	0.980
BMI at 4 y, kg/m^2 †^	15.2(1.9	15.4(2.1)	15.3(1.9)	0.026	0.268

GDM, gestational diabetes mellitus; BMI, body mass index; GWG, gestational weight gain

* Sample size was 6008. ^†^ Sample size was 3285. ^a^The differences in characteristics among three groups; ^b^the differences in characteristics between women with GDM diagnosis < 26 weeks and ≥ 26 weeks groups

### The timing of maternal GDM diagnosis, early GWG and offspring growth

Multivariable-adjusted analysis showed the association of the timing of maternal GDM diagnosis with the growth trajectory in offspring (Table 2). For fetuses, the significant relationships between the exposure to GDM before 26 weeks and an increased Z-score of AC [β coefficient with 95% CI: 0.25(0.18, 0.33)], as well as the higher risk of AC overgrowth [OR with 95% CI: 1.19(1.04,1.36)], were observed when compared with unexposed. Similarly, maternal GDM diagnosis before 26 weeks was associated with increased Z-score of length [β coefficient with 95%CI: 0.09(0.01, 0.18)] and weight [β coefficient with 95%CI: 0.10(0.01,0.18)] in offspring at birth. Moreover, we found the association of GDM diagnosed before 26 weeks with higher risks of LGA [OR with 95%CI: 1.51(1.19,1.91)] and HC overgrowth [OR with 95%CI: 1.32(1.01,1.73)]. Early maternal GDM diagnosis was positively associated with the magnitude of infant BMI peak [β coefficient with 95%CI: 0.16(0.03,0.28)] as well as prepeak BMI velocity of infant BMI peak [β coefficient with 95%CI: 0.04(0.01,0.07)] and was inversely associated with the age of infant BMI peak [β coefficient with 95%CI: -0.16(-0.31, -0.01)] when compared with unexposed (Table 2). In addition, infants exposed to the GDM diagnosis < 26 weeks had higher prepeak BMI velocity, BMI peak, as well as the earlier age of BMI peak (Table 2). Interestingly, these significant associations between GDM diagnosed after 26 weeks and offspring growth were not observed.

**Table 2:** The association of gestational age at GDM diagnosis, maternal early GWG and offspring development

Parameters	GDM < 26 weeks vs. unexposed	*P* value			GDM ≥ 26 weeks vs. unexposed	*P* value		GWG	*P* value	
**Fetus** ^a^										
**BPD**										
Z-score (β with 95% CI)	-0.02(-0.12,0.04)	0.412		-0.03(-0.10,0.04)		0.413		0.51(0.25,0.76)	< 0.001	
Overgrowth (OR with 95% CI)	0.99(0.68,1.32)	0.880		0.99(0.72,1.36)		0.758		1.28(1.01,1.62)	0.042	
**AC**										
Z-score (β with 95% CI)	0.25(0.18,0.33)	< 0.001		-0.07(-0.16,0.02)		0.136		0.64(0.45,0.82)	< 0.001	
Overgrowth (OR with 95% CI)	1.19(1.04,1.36)	0.010		0.96(0.81,1.14)		0.629		1.35(1.20,1.52)	< 0.001	
**FL**										
Z-score (β with 95% CI)	0.003(-0.04,0.05)	0.938		-0.06(-0.15,0.04)		0.248		0.62(0.26,0.98)	0.001	
Overgrowth (OR with 95% CI)	0.96(0.80,1.15)	0.658		1.02(0.88,1.19)		0.980		1.24(1.10,1.40)	0.001	
**Newborn** ^b^										
**Length**										
Z-score (β with 95% CI)	0.09(0.01,0.18)	0.032		0.10(-0.01,0.20)		0.066		0.61(0.47,0.75)	< 0.001	
Overgrowth (OR with 95% CI)	1.21(0.95,1.53)	0.121		1.27(0.96,1.69)		0.092		1.50(1.30,1.75)	< 0.001	
**Weight**										
Z-score (β with 95% CI)	0.10(0.01,0.18)	0.023		0.08(-0.02,0.18)		0.100		0.83(0.70,0.96)	< 0.001	
LGA (OR with 95% CI)	1.51(1.19,1.91)	0.001		1.29(0.96,1.72)		0.087		2.16(1.85,2.51)	< 0.001	
**HC**										
Z-score (β with 95% CI)	0.10(-0.02,0.21)	0.088		0.08(-0.06,0.22)		0.258		0.60(0.42,0.79)	< 0.001	
Overgrowth (OR with 95% CI)	1.32(1.01,1.73)	0.039		1.35(0.98,1.86)		0.062		1.51(1.26,1.81)	< 0.001	
**Infant** ^c^										
Age at BMI peak (β with 95% CI)	-0.16(-0.31,-0.01)	0.038		0.09(-0.10,0.29)		0.342		0.05(-0.23,0.33)	0.748	
Magnitude at BMI peak (β with 95% CI)	0.16(0.03,0.28)	0.016		-0.16(-0.32,0.01)		0.063		0.60(0.36,0.84)	< 0.001	
Prepeak BMI velocity (β with 95% CI)	0.04(0.01,0.07)	0.005		-0.01(-0.05,0.03)		0.636		-0.02(-0.08,0.03)	0.412	

BPD, bi-parietal diameter; GWG, gestational weight gain; AC, abdominal circumference; FL, femur length; HC, head circumference

^a^Adjusted for maternal age, education, household income, pre-pregnancy BMI, early GWG rate, family history of diabetes, parity and the supplement of nutrients

^b^Adjusted for maternal age, education, household income, pre-pregnancy BMI, early GWG rate, family history of diabetes, parity, the supplement of nutrients and mode of delivery

^c^Adjusted for maternal age, education, household income, pre-pregnancy BMI, early GWG rate, family history of diabetes, parity, the supplement of nutrients, as well as mode of delivery, and sex

After adjusting several of confounders, early GWG rate was positively associated with increases in Z-score of fetal ultrasound measurements [β coefficient with 95%*CI* for BPD: 0.51(0.25,0.76); β coefficient with 95%CI for AC: 0.64(0.45,0.82); β coefficient with 95%CI for FL: 0.62(0.26,0.98)], and Z-scores of newborn outcomes [β coefficient with 95%*CI* for length: 0.61(0.47,0.75); for weight: 0.83(0.70,0.96); for HC: 0.60(0.42,0.79)], as well as magnitude of infant BMI peak [β coefficient with 95%*CI*: 0.60(0.36,0.84)]. The significant associations of maternal early EGWG with the higher risks of overgrowth in utero [OR with 95%*CI* for BPD: 1.30(1.03,1.65); for AC: 1.35(1.20,1.52); for FL: 1.24(1.10,1.40)] and at birth [OR with 95%*CI* for length: 1.50(1.30,1.75); for HC: 1.51(1.26,1.81)], for LGA: 2.16(1.85,2.51)] (Table 2) were observed.

### The associations of maternal hsCRP with offspring growth

We also explored the associations of maternal hsCRP with offspring growth (Table S2). The positive relationship between maternal hsCRP levels and increased Z-scores of neonatal outcomes [β coefficient with 95%*CI* for weight: 0.18(0.08, 0.27); β coefficient with 95%*CI* for HC: 0.23(0.10,0.36)] as well as magnitude of infant BMI peak [β coefficient with 95%*CI*: 0.17(0.01,0.33)]. In addition, we found the correlation between maternal higher levels of hsCRP and an increased risk of neonatal HC overgrowth [OR with 95%*CI*: 1.57(1.19,2.07)].

### Interrelationships among GDM diagnosis combined with early EGWG, maternal hsCRP and offspring growth

**Fig. 1:**
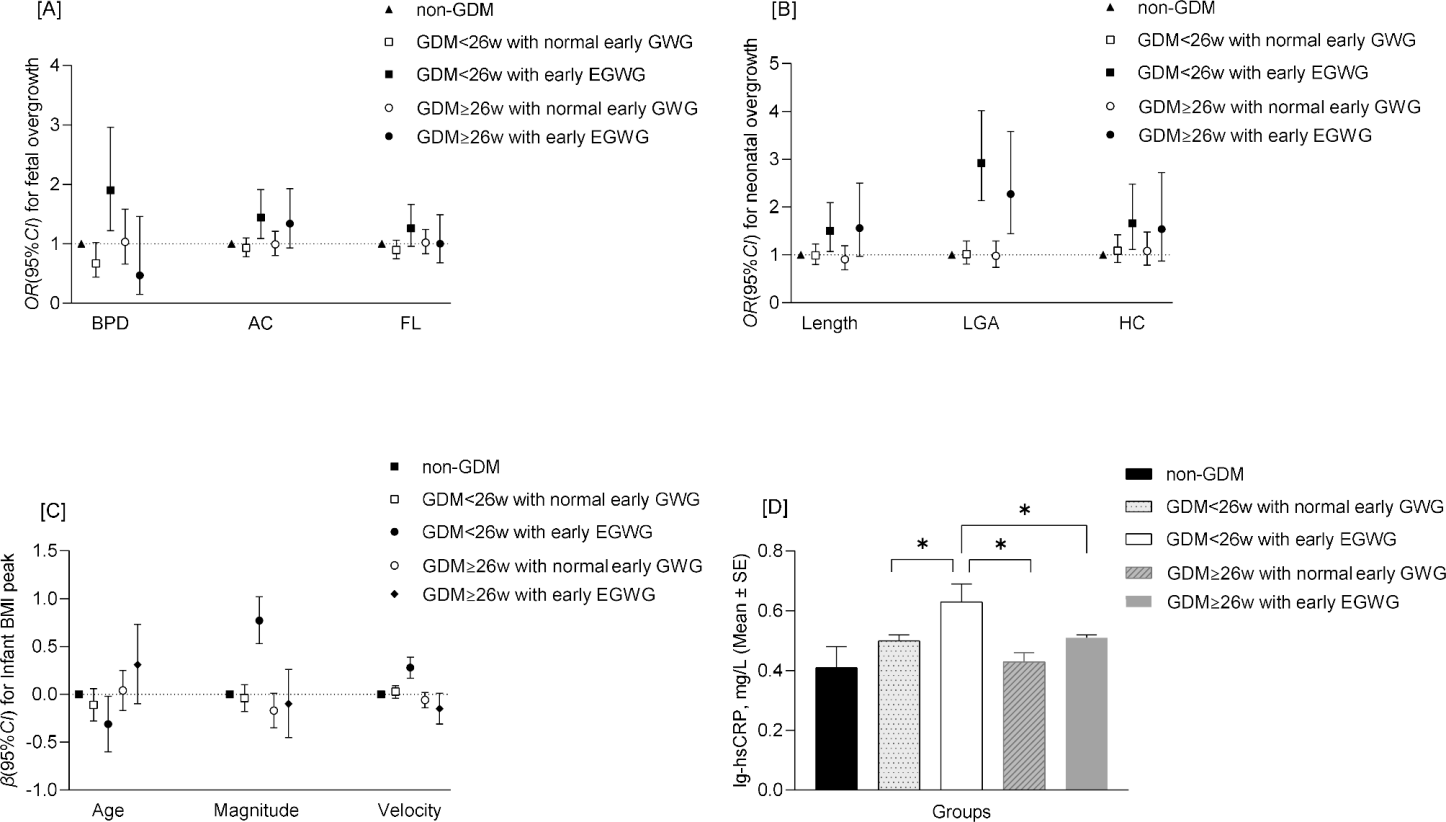
The associations of gestational age at gestational diabetes mellitus diagnosis and early excessive gestational weight gains with offspring development and maternal high-sensitivity CRP. (**A**), (**B**) and (**C**) Associations of the timing of GDM diagnosis and GWG with offspring overgrowth from perinatal to infancy. (**D**) The differences of hsCRP (log10-transformed) among 5 groups (non-GDM and non-EGWG, GDM < 26 weeks with normal early GWG, GDM < 26 weeks with early EGWG, GDM ≥ 26 weeks with normal early GWG, and GDM ≥ 26 weeks with early EGWG.). (**A**) adjusted for maternal age, education, household income, pre-pregnancy BMI, family history of diabetes, parity, and the supplement of nutrients. (**B**) adjusted for maternal age, education, household income, pre-pregnancy BMI, family history of diabetes, parity, the supplement of nutrients and mode of delivery. (**C**) adjusted for maternal age, education, household income, pre-pregnancy BMI, family history of diabetes, parity, the supplement of nutrients and mode of delivery. (**D**) adjusted for maternal age, education, household income, pre-pregnancy BMI, family history of diabetes, parity, and the supplement of nutrients. BPD, bi-parietal diameter; AC, abdominal circumference; FL, femur length; HC, head. circumference; EGWG, excessive gestational weight gain

Stratified by gestational age at GDM diagnosis and maternal early EGWG, the study population was divided into 5 groups: non-GDM and non-EGWG, GDM < 26 weeks with normal GWG, GDM < 26 weeks with EGWG, GDM ≥ 26 weeks with normal GWG, and GDM ≥ 26 weeks with EGWG. Compared with non-GDM group, offspring in GDM < 26 weeks with excessive GWG group had higher risk of overgrowth in utero and at birth (Fig. 1A and B). Subsequently, infants in the GDM < 26weeks with early EGWG had increases in the indicators of infant BMI peak [β coefficient with 95%*CI* for magnitude: 0.77(0.53,1.02); for prepeak BMI velocity: 0.11(0.05,0.16)] and had a significant decrease in age of infant BMI peak [β coefficient with 95%*CI*: -0.31(-0.59, -0.02)] (Fig. 1C). Further, the differences in maternal hsCRP (log-10 transformed) among 5 groups were showed and women in GDM < 26 weeks with early EGWG group had the higher levels of hsCRP (*P* < 0.001) (Fig. 1D).

## Discussion

In this prospective birth cohort study, we found the significant associations of maternal GDM diagnosed by 26 weeks’ gestation with fetal overgrowth for AC in utero, increased weight at birth and the higher BMI peak during infancy compared with GDM diagnosed after 26 weeks’ gestation. Moreover, the current study has demonstrated the additive impacts of maternal early GDM diagnosis and EGWG on the shifts of growth trajectory in offspring. These differences could be explained in part by high levels of maternal antenatal hsCRP. Therefore, these results suggested that increases in maternal inflammatory responses during pregnancy exert the potential impacts on the relationship between intrauterine exposure to metabolic disorders early during pregnancy and elevated risks of later obesity in offspring.

Previous studies have found that early screening for GDM is recommended for women with one or multiple risk factors such as previous gestational diabetes and higher FPG compared with those who received late screening for GDM [27]. Furthermore, women who suffered from hyperglycemia by 20 weeks’ gestation have an increased risk of fetal overgrowth and higher perinatal mortality [4]. In our study, women with GDM diagnosis before 26 weeks’ gestation have higher early GWG rate and fast plasma glucose, and were more multiparous compared with those with late diagnosis of GDM. Tests earlier than recommended timing of GDM diagnosis in pregnancy could be a proxy of early onset of GDM. However, some of women with late screening for GDM could be misclassified because the true onset of GDM may occur early in pregnancy prior to GDM diagnosis. Therefore, our results suggested that control of early EGWG and glucose levels earlier than 26 weeks might be crucial in decreasing overgrowth risk in offspring.

Few previous studies reported the relationship between the time of GDM diagnosis and growth in offspring [4, 5]. Evidence from the Brain Child Study showed that maternal GDM by 26 weeks’ gestation was associated with the increased waist-to-height ratios, whereas GDM diagnosed > 26 weeks’ gestation was not [5]. Another prospective cohort study found that accelerated growth of fetal AC could be identified prior to the diagnosis of GDM [4]. In line with these results, the present study has demonstrated the association of maternal GDM by 26 weeks’ gestation with the increased risks of AC > 90th percentile and the higher BMI peak in offspring when compared with non-GDM mothers. Although detailed information on the obesity outcomes in childhood was unavailable in our study, the BMI peak based on the repeated measures in infancy has been recognized as an important predictor the later obesity [8]. Together, this is the first report on the impact of timing of early maternal GDM diagnosis on the abnormal growth trajectory from perinatal to infancy in offspring.

Excessive GWG beyond the IOM guidelines was related to maternal and neonatal complications such as maternal cardiovascular diseases and LGA [10, 28]. Furthermore, women with EGWG at first presentation with GDM had the higher risk of an LGA infant than women without EGWG [29]. A small-scale study found that early EGWG was associated with greater body fat in newborns [9]. In the present study, our data showed slightly additive impacts of maternal GDM by 26 weeks’ gestation and early EGWG on the overgrowth for fetal AC, the larger birth sizes and higher infancy BMI peak as well as velocity in offspring. These evidence indicated that prevention of early EGWG was recommended before conception and was addressed early in pregnancy. Interestingly, several RCT have confirmed that limiting GWG and treatment for GDM have a modest effect on the short-term outcomes in offspring [30, 31]. Nevertheless, the effect on outcomes beyond the postpartum period and even childhood is ambiguous. Thus, we provided new evidence on the significant associations of maternal early GDM screening and early EGWG with accelerated growth during infancy based on this cohort study, which might predict the risks of later obesity status in children.

Low-grade chronic inflammation is a potential mechanism linking maternal metabolic complications with the development in offspring [12, 13]. A series of studies have used hs-CRP as one of the recognized inflammatory biomarkers, which is characterized as a relatively longer half-life and a stable assessment of the inflammatory state among pregnant women [32]. In the present study, women who were diagnosed as GDM by 26 weeks’ gestation combined with EGWG have significantly higher levels of hsCRP than women diagnosed as GDM after 26 weeks’ gestation. The subsequent analysis also found that children exposed to the higher levels of maternal hsCRP during pregnancy have a higher infancy BMI peak. These data suggested that increased inflammatory responses could mediate the association of the GDM and EGWG exposure early during pregnancy with the shifts and/or disruptions from the typical growth trajectory. In addition, reducing maternal inflammation levels could be an effective strategy for the prevention of adverse outcomes in children like diet intervention. Higher anti-inflammatory potential of maternal diet pattern was related to decreasing risks of GDM and optimal growth of children [33, 34].

Several strengths exist in this study. It is the first report to estimate the interrelationship among the time of maternal GDM diagnosis, EGWG and the growth trajectory of fetal ultrasound measurements, birth size and infancy BMI based on this large-scale prospective cohort study. Of note, multiple measures of early life growth in the current study were used for estimating the infancy BMI growth, which predicted the later obesity during childhood and even adolescence. A further strength was that we performed the potential impacts of maternal inflammatory responses during pregnancy on these relationships. Finally, we have adjusted a series of confounders from pre-pregnancy to the postpartum period to ensure the stability of our results.

However, there are some limitations in our study. Causality cannot be definitively established from a single observational study. We cannot exclude the possibility that women with an early onset of GDM undergo a late test of OGTT due to (likely influenced by healthcare utilization and other external factors) and those women would be misclassified. Considering this condition, the impact of early GDM diagnosis on infants’ growth could be underestimated. Detailed information on later obesity outcomes was unavailable in our study although infancy BMI growth was applied in predicting the later growth in childhood or adolescence but we have demonstrated that maternal GDM diagnosed by 26 weeks’ gestation was associated with the higher BMI in early childhood. Moreover, we have no access to body composition information such as body fat and lean mass both in neonates and infants related to the adiposity. We did not consider biases that may occur at different points in the study, particularly through the exclusion of infants who did not have the minimum number of measurements of weight and length. The level of hsCRP, a single indicator of maternal inflammation was applied in the study, which could not represent the integral inflammatory status.

## Conclusion

In summary, our results indicated the associations of maternal GDM early during pregnancy and EGWG at the first presentation of GDM with excessive growth in offspring from perinatal to infancy. Increased levels of maternal antenatal inflammation could represent a potential biological mechanism how prenatal metabolic disorders exert detrimental impacts on offspring’s overgrowth. Both screening for GDM and healthy maternal weight gain earlier in pregnancy are essential to shaping the optimal growth trajectory for preventing and intervening the later overweight or obesity.

### Electronic supplementary material

Below is the link to the electronic supplementary material.

Supplementary Material 1

## Data Availability

The datasets used and/or analyzed during the current study are available from the corresponding author on reasonable request.

## Abbreviations

GDM: gestational diabetes mellitus
EGWG: excessive gestational weight gains
LGA: large for gestational age
hsCRP: high-sensitivity C-reactive proteins
