# Supplementary material for: Timing of gestational diabetes diagnosis, gestational weight gains and offspring growth trajectory: a prospective birth cohort study
Source: BMC Pregnancy Childbirth. 2023 Sep 7;23:642. doi: 10.1186/s12884-023-05954-2 (PMC10483803; doi:10.1186/s12884-023-05954-2)
Supplement: Supplementary file 1 — Supplementary Material 1 [file 12884_2023_5954_MOESM1_ESM.pdf]

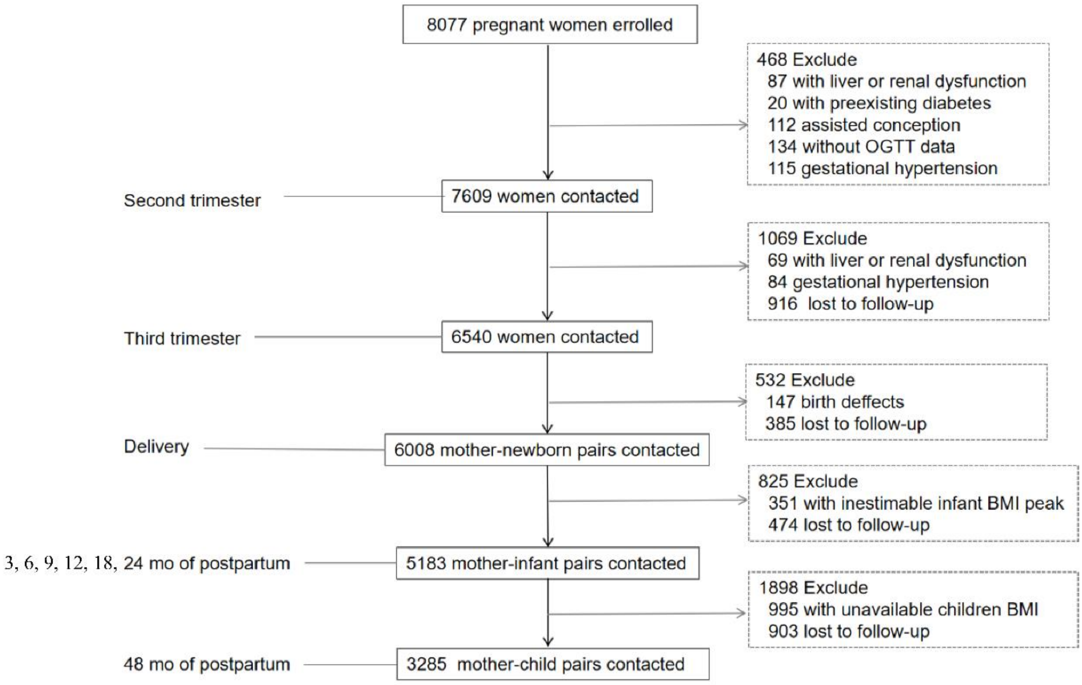

Figure S1. Flowchart illustrating enrolment for MIH study

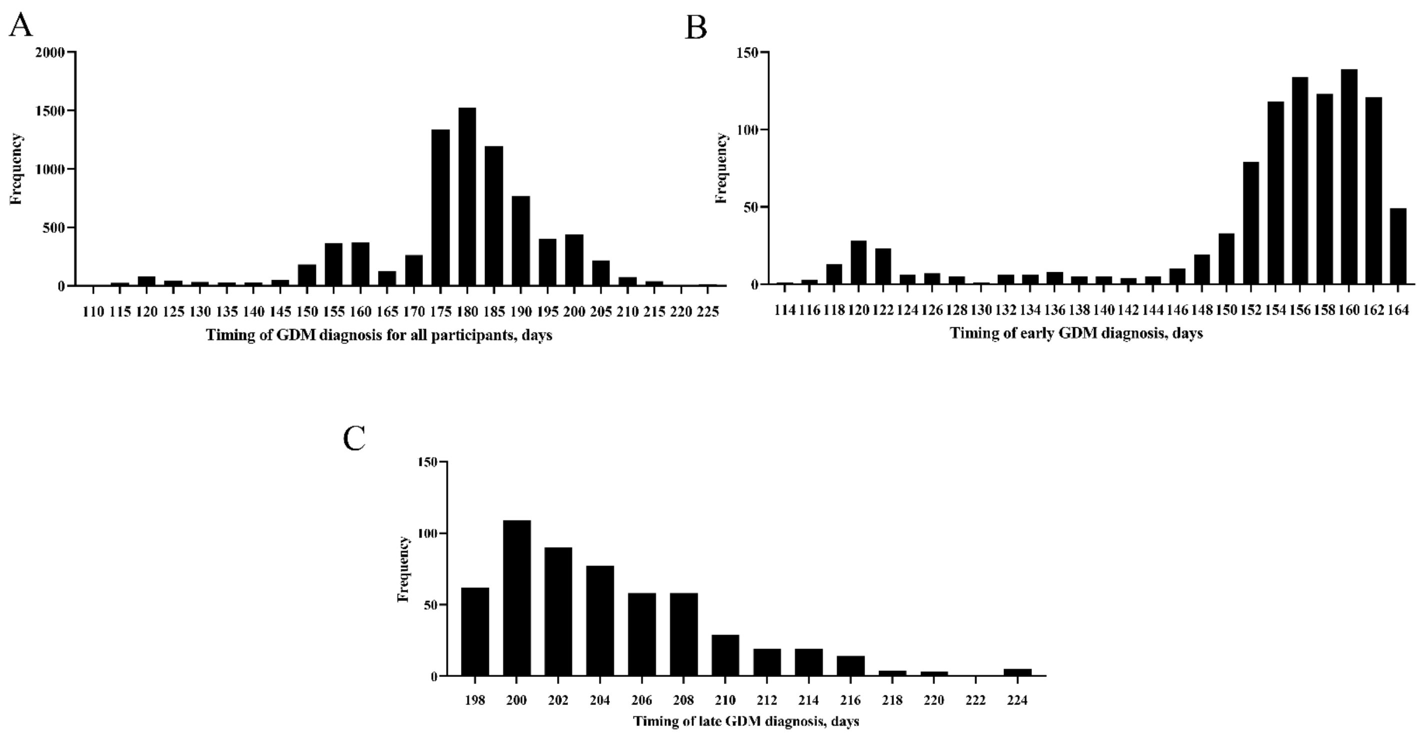

Figure S2. The distribution of the timing of GDM diagnosis

Table S1. Characteristics of the participants versus non-participant in the study.

Characteristics	Participant N=3285	Non-participant N=4324	*P* value
**Mother**			
Age, y	29.17(3.50)	29.05(4.85)	0.152
Prepregnancy BMI, kg/m^2	21.52(2.75)	21.61(3.08)	0.188
Education ≤ 12 y	2470(75.2)	3252(75.2)	0.986
Household income < 4000 yuan	680(20.7)	774(17.9)	0.002
Multiparous	1269(38.6)	1704(39.4)	0.517
Early GWG rate, kg/wk	0.30(0.16)	0.29(0.17)	0.169
Late GWG rate, kg/wk	0.52(0.31)	0.52(0.34)	0.698
Total GWG rate, kg/wk	0.38(0.13)	0.38(0.13)	0.231
Family history of diabetes	322(9.8)	402(9.3)	0.500
Physical exercise ≥ 3days/wk	1376(41.9)	1877(43.4)	0.198
Folic acid supplement <1 times/d	2516(76.6)	3325(76.9)	0.754
Calcium supplement <1 times/d	854(26.0)	1090(25.2)	0.408
Iron supplement <1 times/d	198(6.0)	29(6.8)	0.151
**Infant**#			
Birth weight, kg	3.40(0.44)	3.40(0.47)	0.076
Delivery gestational age, week	39.37(1.20)	39.36(1.43)	0.688
Male sex	1546(52.8)	1896(52.2)	0.657
Vaginal delivery	1935(66.1)	2418(66.6)	0.668

Sample size of participants was 2929, sample size of non-participants was 3630.

Table S2. The association of maternal hsCRP and offspring development.

Parameters	lg-hsCRP	
	β/*OR* (95%*CI*)	*P* value
**Fetus** [a]		
**BPD**		
Z-score	-0.03(-0.13,0.07)	0.512
Overgrowth	0.87(0.58,1.31)	0.509
**AC**		
Z-score	-0.02(-0.16,0.12)	0.781
Overgrowth	1.02(0.85,1.24)	0.808
**FL**		
Z-score	-0.05(-0.16,0.06)	0.348
Overgrowth	0.97(0.80,1.17)	0.735
**Newborn** [b]		
**Length**		
Z-score	0.07(-0.03,0.17)	0.158
Overgrowth	0.81(0.62,1.05)	0.103
**Weight**		
Z-score	0.18(0.08, 0.27)	<0.001
LGA	1.12(0.87, 1.44)	0.395
**HC**		
Z-score	0.23(0.10,0.36)	<0.001
Overgrowth	1.57(1.19,2.07)	0.016
**Infant** [c]		
Age at BMI peak	0.05(-0.14,0.23)	0.623
Magnitude at BMI peak	0.17(0.01,0.33)	0.038
Prepeak BMI velocity	-0.01(-0.05,0.03)	0.785

Adjusted for maternal age, education, household income, prepregnancy BMI, total GWG rate, family history of diabetes, parity, the supplement of nutrients, as well as mode of delivery.
